# Perceived stigma, substance use and self-medication in night-shift healthcare workers: a qualitative study

**DOI:** 10.1186/s12913-022-08018-x

**Published:** 2022-05-24

**Authors:** Lorraine Cousin, Guillaume Roucoux, Anne Sophie Petit, Laurence Baumann-Coblentz, Olivia Rousset Torrente, Adriano Cannafarina, Olivier Chassany, Martin Duracinsky, Patrizia Carrieri

**Affiliations:** 1 Unité de Recherche Clinique en Economie de La Santé (URC-ECO), AP-HP, Hôpital Hôtel-Dieu, F-75004 Paris, France; 2 Patient-Reported Outcomes Unit (PROQOL), UMRS 1123, Université Paris Cité, INSERM, F-75004 Paris, France; 3 Groupe de Recherche en Psychologie Sociale (UR GRePS), Université Lyon 2, Bron, France; 4 Département de médecine générale, Université de Paris, F-75010 Paris, France; 5 Département de Médecine Interne Et d’immunologie Clinique, Hôpital Bicêtre, AP-HP, 94275 Kremlin Bicêtre, France; 6 Aix Marseille Univ, Inserm, IRD, SESSTIM, Sciences Economiques & Sociales de la Santé et Traitement de l’Information Médicale, ISSPAM, Marseille, France

**Keywords:** Qualitative study, Addiction, Health workers, Shiftwork, Occupational health

## Abstract

**Background:**

Many risk factors related to altered circadian rhythms impact the health of night-shift hospital workers (NSHW), resulting in mental and somatic disorders. Easy access to psychoactive substances (PS) may facilitate addictive behaviors in NSHW. They are also exposed to a stressful work environment, which may further affect sleep quality. This study aimed to explore the link between sleep deprivation, work-related psychosocial stress and psychoactive substance use as a self-medication response in NSHW.

**Methods:**

Qualitative study to verify the plausibility of the self-medication theory applied to addictive behaviors. Semi-structured interviews (*N* = 18 NSHW) and thematic analysis, following consolidated criteria for reporting qualitative research recommendations.

**Results:**

Stigma against NSHW was a primary element of a stressful work environment. The stressful and stigmatizing environment, together with night-shift work, further affected NSHW sleep and their mental and physical health. The use of PS appeared to be for self-medication, encouraged by social and professional environments, source(s) of stress, discrimination, and isolation. The work environment, through aggravated sleep disorders, led NSHW to use non-prescribed sleeping pills. Alcohol after work and smoking were used as a social break but also as a means to reduce stress.

**Conclusion:**

Anti-stigma interventions in the healthcare setting and screening of mental/somatic disorders in NSHW can help reduce harmful self-medication behaviors and improve hospital care in the COVID-19 era.

**Supplementary Information:**

The online version contains supplementary material available at 10.1186/s12913-022-08018-x.

## Background

The ongoing COVID-19 health crisis highlights the indispensable contribution which health professionals provide to society and the importance of their quality of working life (QWL) in ensuring a high standard of care during this prolonged crisis period [1–4].

Night-shift workers are exposed to specific health risks caused by altered circadian rhythms [5, 6]. They suffer from sleep disorders, nutritional imbalance, increased cardiovascular risk and metabolic syndrome, and are at greater risk of breast cancer and disorders during pregnancy leading to low birth weight and miscarriage [7, 8]. Poor sleep quality in night-shift healthcare workers (NSHW) is strongly correlated to psychological and somatic symptoms, such as mood disorders or increased anxiety and irritability. Night-shift work may also lead to disruptions in social and family life [9], poor mental quality of life [8], reduced leisure time, poor psychological well-being [9], and less social support [10]. All these elements are risk factors for psychoactive substance (PS) use, and are a source of QWL deterioration [11, 12].

Easy access to PS may facilitate substance use in NSHW [13]. Moreover, structural factors related to work organization (e.g., understaffing) constitute additional sources of work-based stress. Similarly, contextual factors (e.g., worsening of pulmonary pathologies observed more frequently during the night) [14] have been shown to increase stress in NSHW related to their sense of responsibility towards their patients.

Exposure to stress increases PS self-administration [15]. Moreover, chronic PS use—by altering the activity of the brain—can reduce reactivity to stress [16], in turn leading to addiction. According to Khantzian’s [17] self-medication hypothesis (SMH), the use of PS occurs in a context of self-regulation of the psychological difficulties which users face, such as stress, impaired self-esteem and altered relationships [18]. This hypothesis was originally based on persons who used heroin to reduce dysphoria. Later, it was extended to the use of different PS, such has alcohol and cocaine, in order to manage psychological dimensions such as stress and depression. We can therefore hypothesize that PS consumption by NSHW may be an attempt to self-manage stress, work rhythms (understood here as night-shift work, shift duration and workload), and sleep disorders, as well as their consequences. This ‘self-medication’ behavior is not without risk for hospital staff or indeed their patients [19], since it can lead to decreased alertness. No study to date in France has ever explored PS use in NSHW, and only a few studies internationally have investigated this question, despite the fact that this population is at a higher risk of consumption [20, 21], and frequently this is for self-medication.

### Aim

This qualitative study aimed to investigate how sleep deprivation and work-related psychosocial stress impact NSHW use of PS.

## Methods

### Participants

Eighteen semi-structured interviews were conducted with full-time NSHW who worked night shifts at least twice a week in 10 *Assistance Publique–Hôpitaux de Paris* (AP-HP) hospitals. The AP-HP network is the largest healthcare institution in France, employing 100,000 professionals in the *Ile de France* region alone. This large number of professionals and their diversity make the network an invaluable study setting to better understand the structural mechanisms acting on the health of NSHW. Persons working at least two night shifts a week, working exclusively at night, or alternating between day and night shifts in an AP-HP hospital were eligible to participate. In order to keep the study population homogeneous, physicians were excluded from the analyses as they constitute a subgroup with specific characteristics. To achieve maximum variation sampling, several different types of NSHW were included: nurses, nurse assistants, x-ray technicians, lab technicians, midwives, child-care assistants, and health managers. Among the 18 participants, 17 worked only night shifts and one alternated between day and night shifts. Seventeen worked a 10- or 12-h shift, and one an 8-h night-shift. The study used purposive and theoretical sampling, whereby emerging themes are further explored by deliberately seeking new participants with characteristics that could expand or challenge the theme currently being discussed. The purposive sample needed to be diverse in terms of age, gender, occupation, and the hospital departments in which the participants worked. Furthermore, we wished to have as wide a variety as possible in terms of participant experience, background, work practice, and organization. To achieve this, the first participants recruited were mostly men who worked in general departments. In the second round of recruitment, more women were recruited. The final third round focused on recruiting older participants. New participants were enrolled using snowball sampling. Participation was spontaneous with workers meeting the investigator during the latter’s visits to hospital wards or by e-mail. In accordance with the general inductive approach, the number of participants required was not predefined; recruitment was stopped when data saturation was reached (i.e., when no new information emerged, and when a basic social process had been identified [22]).

### Settings

Semi-structured face-to-face interviews were performed using an ad hoc guide (Annex 1) (16 individual interviews and one dyadic interview (i.e. with two participants) [23]). Interviews lasted between 35 to 45 min and took place in participants’ homes or their hospital workplace. All interviews were recorded and transcribed for analysis. The guide used was constructed by a seven-person scientific committee comprising two addiction specialists, one nurse, three researchers, and one occupational physician. Its contents were based on the latest scientific literature. The committee identified the topics to explore in the guide through brainstorming sessions based on the research question, the study objectives, and related literature. The research question was constructed based on a literature review performed by the team’s researcher in public health (LC). Three versions of the interview guide were produced and submitted to the committee to prioritize and refine the topics to be investigated, the focus being on establishing how and when PS was used by NSHW, identifying what PS they used, and examining how PS use was related to the negative effects of their work environment. Accordingly, the topics chosen were the organization of night-shift work, relationships with colleagues, relationships with family and friends, health, and PS use (Annex 1).

### Regulatory and ethical aspects

This study was approved by the INSERM Ethics Committee (CEEI CD/EB 20–005). Consent was obtained from each participant before being interviewed. All data are confidential and anonymous.

### Analysis

Data were collected according to the general inductive approach [24] and thematically analyzed using NVIVO 11 software. The study was conducted in accordance with consolidated criteria for reporting qualitative research recommendations (COREQ) [25]. First, three experienced researchers in thematic analysis (an ethno-sociologist (GR), a senior researcher specialized in qualitative studies (MD), and a researcher in public health (LC)) analyzed the collected data in order to explore individual experiences of being an NSHW, and to compare documented experiences between different NSHW. Second, the investigators systematically identified the themes which emerged in each interview, that is to say, the different units of meaning that had a sense for the interviewees [26]. Finally, the themes and units of meaning were triangulated. During the triangulation sessions, each investigator individually pooled and coded the themes. Coded themes were then compared and discussed by all the investigators to reach consensus.

The experiences and narratives of the participants allowed us to describe the phenomena and mechanisms of substance use in this NSHW sample using a comprehensive approach. This thematic analysis allowed us to highlight consumption practices and to investigate PS consumption issues in NSHW in greater detail. The analyses involved data reduction, followed by data display, and then conclusion. Discussions between the investigators led to the construction of a matrix and a thematic tree.

## Results

### Study population

This qualitative study was conducted between October 2019 and February 2020. Twenty-five professionals were contacted. Of these, five did not respond, and two did not agree to have their discourses audio recorded. Among the 18 remaining NSHW who agreed to participate and be recorded, eleven were men and seven women. Median age was 32 years and years of work experience varied between 2 and 29 years. Six participants were nurses, three nurse assistants, two X-ray technicians, two laboratory technicians, one midwife, one childcare assistant, and three department managers. The latter were supervisors of paramedical teams who worked at night. Seventeen worked only night shifts, and one participant alternated between day and night shifts. Seventeen worked a 10- or 12-h shift, and one an 8-h nightshift. Table 1 displays the study sample’s characteristics.

**Table 1:** Participants’ characteristics

Pseudonym	Status	Number of Children	Work	Gender	Age	Department	Number of years of work experience (yrs)	Number of years working night shifts (yrs)	Shift length (hours)	Shift type
H1	Single	0	Nurse	M	27	General ward	5	3	10	Ex-night worker
H2	Single	0	X-ray technician	M	33	General ward	5	5	12	Night worker
H3	Single	0	X-ray technician	M	31	General ward	8	8	12	Night worker
F1	Single	0	Nurse	F	25	General ward	3	2,5	10	Night worker
H4	In a Relationship	0	Nurse	M	29	A&E^a^	4	4	12	Night worker
F2	In a Relationship	0	Nurse	F	23	A&E	2,5	2,5	12	Night worker
F3	Single	0	Child care assistant	F	25	A&E	2	2	12	Night worker
H5	In a Relationship	0	Nurse-assistant	M	56	A&E	28	21	10	Night worker
H6	In a Relationship	5	Nurse-assistant	M	50	General ward	15	15	10	Night worker
H7	Married	0	Health manager	M	51	A&E	29	20	12	Night worker
F4	In a Relationship	0	Midwife	F	28	General ward	3,5	3, 5	12	Alternate night/days
F5	Single	0	Nurse	F	29	General ward	4	0,1	10	Night worker
F6	Single	1	Nurse	F	47	General ward	20	20	10	Night worker
H8	In a Relationship	0	Lab technician	M	24	Lab	4	3,5	12	Night worker
H9	Married	0	Lab technician	M	51	Lab	20	18	12	Night worker
F7	In a Relationship	3	Health manager	F	39	All services	unknow	6	10	Night worker
H10	In a Relationship	3	Senior health manager	M	45	All services	20	11	8	Night worker
H11	Divorced	3	Nurse-assistant	M	45	A&E	22	13	10	Night worker

^a^*A&E* Accident and emergency department

In terms of age and sex, the most common participant profile was young (i.e., < 35 years old) men. All X-ray technicians and lab technicians interviewed matched this profile. The three managers interviewed were older than the other professionals.

### Thematic analysis results

In the global corpus of 1,432 codes, six themes were identified: (1) Work environment, (2) Private and social life, (3) Health, (4) Sleep and fatigue, (5) Addiction, (6) Ways to improve quality of working life.

The interview guide was adapted after five interviews to integrate themes which had not been initially included in the guide, and which needed more detailed investigation. These emerging themes were: relationship with colleagues and its impact on perception of work, work-related health issues, and difference between day and night shift-work in terms of PS use. All the example quotations from NSHW below depict their representations of themselves, their tasks, and the associations they identified between their work and PS use.

### Work environment: stress, invisibility, stigmatization and autonomy

We identified NSHW environment themes and sub-themes including night-shift only tasks, the calm night-shift environment, a sense of responsibility, health issues, and the importance of private life. NSHW working environment was not conducive to a QWL and could affect consumption practices. Several factors affected NSHW quality of life including negative representations, ambivalence between autonomy and isolation, and inherent stress in night work.

NSHW working tasks differed from those of their daytime counterparts. For example, in some wards, night-shift nurses had fewer technical tasks (e.g., changing bandages), while laboratory technicians had to multi-task as they needed to cover all laboratory posts.

Because of their work rhythms, NSHW were less visible than daytime workers and faced work-related stigmatization. They often saw themselves as being underestimated or stigmatized. All 18 participants reported that other professionals, especially their daytime colleagues, made unpleasant remarks about them and denigrated their work. They also considered that their work was not valued enough by their superiors. This lack of recognition, the stigmatization of their job, and their isolation had an impact on their self-esteem.

*“They say: you don’t do anything at night. Oh yes, they sometime leave us work ‘Anyway you don’t have to do anything at night, you’ll have time to do it’. That’s recurring. They say “you sleep at night, you do nothing…” (F3 – Child care assistant)*

The calm night-shift environment was perceived both positively and negatively; while a small working team could facilitate collaboration, empathy and solidarity, it could also create a feeling of isolation.

Furthermore, the small number of night-shift workers could lead to situations of understaffing and consequent stress, two potentially dangerous factors when facing an emergency. These understaffed situations were less mentioned by health managers and senior managers than by other NSHW.

*
"Eh, and then, what might be more important thing is to be left by yourself, because the slightest problem, you don't have a manager to rely on, it's really, it creates resourcefulness, and it's cool in that way too. […] And well, it’s quite good but at the same time dangerous, I think.” (H2 – X-ray technician)*

Consequently, NSHW felt doubly responsible for their patients and colleagues because of the need to manage emergencies without supervisors. However, these situations also helped improve their sense of autonomy.

The small night-shift team could also increase the risk of accidents when a staff member was not able to work efficiently when he/she was under the effect of PS.

*"[For example] You and another nurse-assistant are on a ward. The other one is drunk or is sleeping in the corner; you’re alone, anything can happen to you, a patient with a problem, anything; you don't have a colleague you can count on; I’ve always found that to be more than dangerous" (H7—department manager).*

### NSHW PS consumption

The PS which NSHW consumed were tobacco/nicotine, alcohol, cannabis, hypnotics, and anxiolytics (Annex 2). The latter two were used as sleeping pills. There was no clear difference between consumption in terms of age, sex or profession. No participant mentioned cocaine, amphetamine, opioids, or intravenous drug use, although all were asked if they did. In terms of tobacco use (smoking cigarettes), four participants were daily smokers, two were regular smokers, and three were former smokers. Alcohol was the most used PS; only one participant reported not drinking. Cannabis use was infrequent. One participant was a former daily cannabis smoker, while three were occasional users. Six participants reported frequently using sleeping pills (melatonin, hypnotics and anxiolytics).

The reasons participants gave for using PS for work and work environment circumstances were stressful situations caused by at-risk situations (e.g., being alone when a patient has convulsions) and feelings of isolation.

Among NSHW who smoked cigarettes, the majority reported they did so to cope with work-related stress. For example, unplanned problems and unmanageable workloads prompted them to take a cigarette break to relax. In addition to relieving stress, cigarette breaks enabled NSHW to talk with colleagues about the difficult situations they encountered. Cigarettes were positively perceived by one department manager, as they improved mood.

*“I also use cigarettes when I’m faced with a really really contrariety because it's a way I've found to relieve stress.” (H7 – department manager)*

Feelings of isolation were a driver for some NSHW to regularly go to parties and social events in order to maintain interpersonal links. This implied binge drinking at least once a week for the youngest interviewees. Some of the respondents reported that their work was a factor which increased their desire to go out to see a friend. Such social events were always synonymous with drinking.

*“Yeah, that's it, an aperitif, a drink at the bar and then that's it […] Yeah, so the abuse was really going out all the time and seeing people, that's what it was.” (H1—nurse)*

One characteristic shared by all the NSHW interviewed was the importance of private life; social life and spare time were essential for them. All but one of the participant reported consuming during their spare time. PS consumption was very different between working hours and non-working hours. More specifically, the substances consumed differed as did the reasons. PS consumption in private life was related to relaxation and pleasure.

**Fig. 1:**
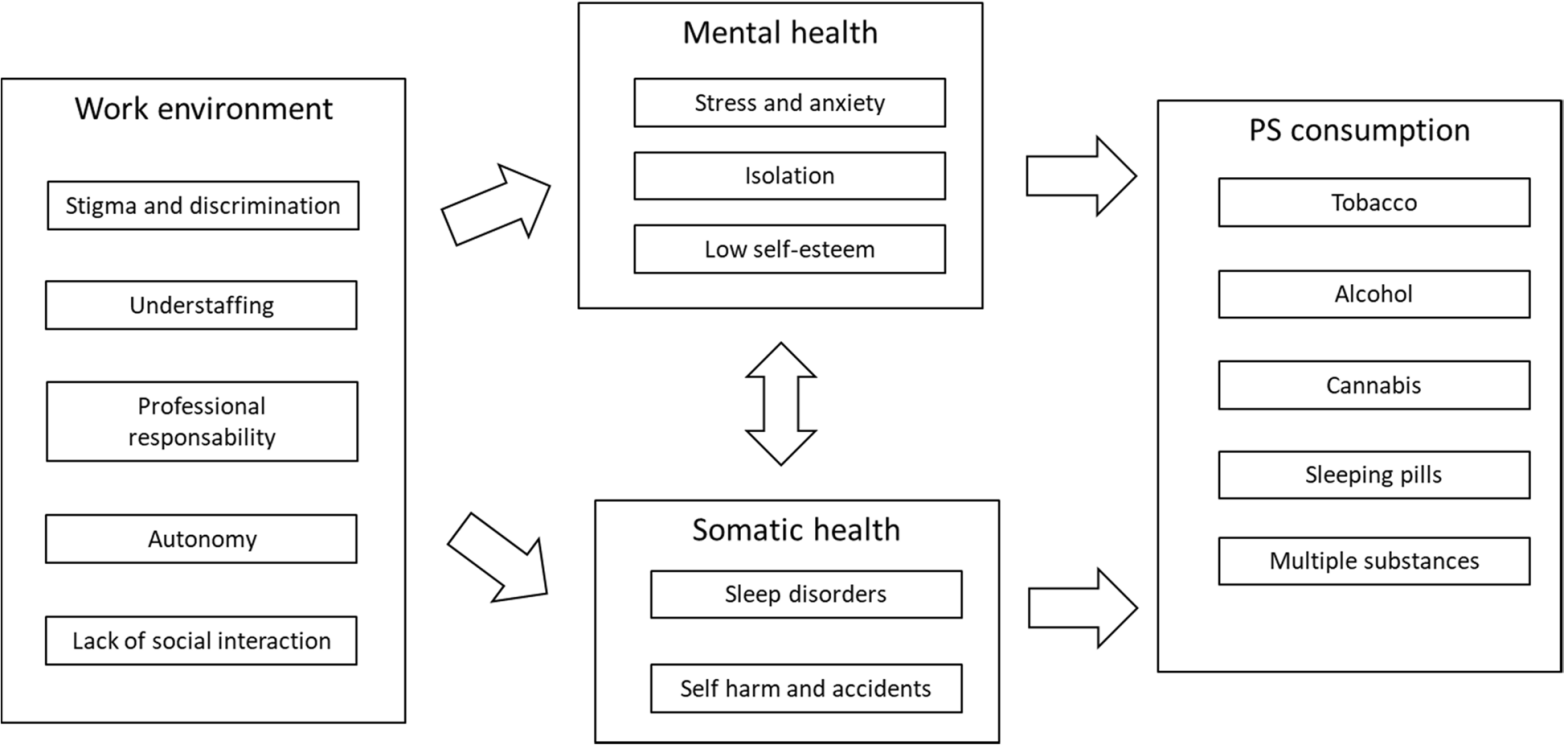
Work environment effect on health and psychoactive consumption

The processes involving the working environment of NSHW and consequences on substance use are explained in Fig. 1.

### The normative aspect of PS consumption: prohibited PS, authorized PS, and related contexts

As previously mentioned, consumption habits differed between work and free-time contexts. Consuming certain PS at work was forbidden (illegal substances and alcohol), although interviewees mentioned alcohol use during work for special events such as farewell parties or Christmas. Smoking breaks and taking medication at work were not forbidden and were socially accepted.

*“For us nurses, for Christmas parties and New Year’s Eve, we just take half a glass, that’s all” (F1—Nurse)*

Consuming cannabis was considered unacceptable by participants because it is illegal, among other reasons. Tobacco smoking was perceived less negatively as it was not prohibited if consumed outside the hospital premises during work breaks. All the participants declared consuming more PS outside of working hours than during work. Outside of work, some smoked tobacco and cannabis, drank alcohol, and took sleeping pills.

The most experienced NSHW reported that in the previous 10 years they had observed a decrease in co-worker acceptability of alcohol and pill consumption in the workplace, as well as a decrease in the number of times they saw their colleagues under the effects of PS while at work.

This evolution corresponds with the strengthening of the normative aspect of consumption in hospitals.

*"At that time, the atmosphere in the hospitals wasn't at all the same, and there were a lot of people using alcohol and medicines, […] at that time, no one hid, I had drunk hospital staff in the workplace, I had staff who actually took medication to keep them going" (H7 – department manager).*

A declared sense of responsibility and awareness of addictive behaviors were factors associated with non-acceptability of PS consumption in the workplace. Non-acceptability of alcohol consumption (apart from farewells and festive events) and cannabis use at work were mostly explained by a sense of professional responsibility. The ban of PS use in the hospital outside of festive occasions governed PS use at work. NSHW reported that they could not afford to consume PS as they would lose concentration, make mistakes and cause accidents.

*"It's more fruit juice and non-alcoholic champagne, because after you’ve got to keep it together. You can't afford to screw up doses and then say to yourself that it's because you were drinking. No, you can't do that. You can’t." (F6—nurse)*

Interviewees were also aware of the consequences of PS consumption on their health and addictive behaviors such as lower concentration and a higher risk of accidents, dangerous for them and for patients. Their theoretical and practical professional knowledge meant they were more likely to question their own consumption of PS. NSHW have both procedural and theoretical knowledge acquired during their training and experiential knowledge acquired with patients suffering from addictions. This knowledge can lead to a personal awareness that has a direct impact on the consumption of PS. For example, a midwife, stopped drinking beer after she realized that she needed it to relax after work. Similarly, three other nurses questioned and reduced their use of alcohol and cannabis when they noticed that it was becoming more frequent and was associated with a need to fall asleep or relax.

*"I'm taking a break from alcohol because I realized that when I came home in the evening it felt good to have a beer" (F4—midwife).*

### Self-medication as a key response to stress and sleep disorders

The study’s NSHW were prone to insomnia and sleep disorders because of the alteration of their circadian rhythms. Some implemented specific lifestyle improvements (e.g., physical activity, psychological therapy) to reduce the impact of work on their sleep. Others used cannabis and/or sleeping pills to manage sleep disorders caused by night-shift work. NHSW looked to the soporific and relaxing effects of cannabis and sleeping pills to help them fall asleep after work, and to help them readjust to daytime rhythms on their days off.

NSHW used various types of sleeping pills. NSHW suffering from insomnia reported that they first used “natural” (such as melatonin and plant) sleeping pills to treat it. If those did not work, they switched to hypnotics and anxiolytics.

*" I tried melatonin, it didn't work; then I tried plant-based pills, that worked for a while but doesn't work so much anymore. So now, I’m on zopiclone® and stuff like that" (F2—nurse)*

In France, the majority of sleeping pills, except melatonin and plant-based pills, require a medical prescription. However, in our study sample none of those who declared the use of hypnotics or anxiolytics had ever received a prescription for these medications. This confirms the hypothesis of self-medication practices. NSHW stated they took sleeping pills directly from the ward’s medicine cabinet. This behavior was not taboo and the respondents mentioned it openly, as they did not see it as something forbidden. They felt that it was tacitly accepted that they could help themselves to these medicines without a prescription.

*“stilnox or imovane, but eh same thing, I didn't take much in the end […] Well, for one thing, it wasn't with a prescription, I lifted some here and there.” (H2 – X-ray technician)*

Indeed, this practice seemed to be so common that one department manager regularly observed missing pills in the hospital where he worked without being able to identify the source. He was troubled by this, because beyond the risk associated with self-medication with anxiolytics and hypnotics, these medicines were intended for patients.

Some NSHW who expressed wanting to quit smoking also mentioned self-medication. They had tried many different methods to quit smoking, some more effective than others, and the most common being e-cigarettes. Some tried nicotine patches or chewing gum, others hypnosis. All these methods had been used for self-medication. That is to say that none were prescribed by a healthcare or addiction professional. In some cases, the specific ways NSHW dealt with their addiction problems and health issues, led to inappropriate use of smoking cessation methods and quit attempt failures.

## Discussion

### Main results

This qualitative study had three main results. First, the NSHW work environment was very stressful because of stigmatization, the need for autonomy, isolation, and at-risk situations. Second, PS use was related to this stressful work environment. For example, smoking was commonly used to manage night-shift work stress, to improve working life, and to reduce the impact of stress on personal life. Third, PS were used for self-medication to try and manage the consequences of sleep disorders and stress.

Working at night therefore had both a direct and indirect effect on the consumption of PS.

### The night-work environment paradox: the desire for working autonomy leads to a feeling of isolation

NSHW measured and compared the level of difficulty of their work against that of daytime workers in terms of tasks, work rhythms and ways of working. They reported that they did not have the same working conditions as their daytime counterparts, and that their work was often underestimated and denigrated. In general, daytime workers are the reference category in studies of hospital workers and NSHW are much less studied because of their invisibility [27]. This invisibility is related to the much greater difficulty to reach NSHW given that their working hours are completely different from the majority of the general population. Elsewhere, NSHW have highlighted the difficulties they face when trying to contact their hierarchy and to find interlocutors who will help them advocate change and improvements in their practice and in their professional lives [28, 29]. At night, work is carried out autonomously, most of the time alone or with a very small number of colleagues and without hierarchical supervision. Although the NSHW in our study sought autonomy, night work generated stress, which in turn fostered PS use. More specifically, NSHW working conditions generate a paradox, because while autonomy is one of the motivations for choosing to work night shifts, this autonomy becomes a burden when unexpected situations require monitoring and supervision outside the scope of NSHW responsibilities.

### NSHW PS use

#### The role of stigmatization

Stigma and discrimination of NSHW results in their work being devalued. Stigma lowers their self-esteem and greatly affects their work [30, 31]. The combination of low self-esteem, which affects mental health, and marginalization of NSHW are risk factors for addiction development [32–34]. The stigmatization of these professionals and their work would therefore seem to be a central element that contributes to the consumption of PS. Actions must be taken to reduce stigmatization in the health setting, for instance using social contact interventions [35].

#### Sleeping pills to counter sleep deprivation

Many studies have demonstrated a strong association between night-shift work and sleep disorders, their prevalence being significantly higher in this population than in daytime workers [19, 36]. These disorders are related to the alteration of circadian rhythms [6] and have been identified as a potential cause of some certain addictive behaviors and psychological distress [37]. In some cases, consuming PS such as alcohol [38] or cannabis [39] may be perceived by NSHW as one solution to counter sleeping disorders. However, using PS in this way may actually worsen the situation, as they do not treat the cause. As mentioned by some participants in the present study, alternative strategies exist. For example, the French National Authority for Health (HAS) recommends behavioral therapy and psychotherapy as first-line treatments for chronic insomnia [40]. Sleep medication (benzodiazepines or hypnotics) should be prescribed only if these therapies do not work adequately, and only in accordance with HAS recommendations [41]. In our study, some NSHW reported skipping alternative treatments, which would have been prescribed by physicians, and directly using pills (melatonin and hypnotics) for their sleeping disorders, because the opportunity to self-medicate was easier. One major difficulty with this, as reported by the same NSHW, is that they did not follow recommendations for use.

#### Tobacco and alcohol for relaxation and social use

In the literature, one risk factor for tobacco use in NSHW is the need to take a break in order to cope with a stressful work environment [42, 43]. Some studies found a relationship between nicotine dependence scores and levels of job stress in nurses who smoked [44, 45]. Similarly, alcohol consumption is also linked to stress at work and to anxiety [46–48]. Moreover, stress is a principal factor in smoking initiation and relapse [49, 50]. In our study, NSHW perceived tobacco and alcohol to be an effective solution to combat anxiety, induce sleep more quickly, to stay awake, and increase their performance [2, 51, 52].

Outside of work, alcohol was consumed as a way to unwind with friends. It was associated with socialization but could lead to dependence, which reflects findings elsewhere [53, 54].

The need to unwind and connect with friends highlighted in our study and elsewhere, may be stronger in NSHW because of the social isolation they face. It is possible that NSHW feel a greater need to belong to a social group and to share activities such as drinking and/or smoking than their daytime working counterparts.

### The heart of the problem: self-medication

The widespread use of self-medication is a key component in problematic PS use in hospital workers [55–57]. Self-medication is present in many different countries and in different medical professions. Paramedics, doctors and dentists are known to self-medicate [38, 39]. It is used to treat a wide range of conditions and for many different reasons (e.g., supposed knowledge of medication, mild sickness, lack of time to consult a professional). In France, self-medication is amplified by the availability of medications in the workplace that cannot be found outside hospitals without a prescription.

In our study, by not consulting with colleagues or other doctors or health professionals outside their workplace, study participants who self-medicated prevented themselves from finding effective solutions to problems they faced, including tobacco cessation attempts and management of their sleep disorders. Self-medication was a common problem among the NSHW interviewed. Tailored interventions are needed to address this issue and decrease PS use in this population.

Self-medication does not only concern the use of medication without medical advice. In accordance with Khantzian’s SMH and Duncan’s work [58], self-medication in our study meant the use of PS to compensate for psychological or social difficulties, and for stigmatizing attitudes affecting self-esteem encountered in the context of and because of work. The SMH is thus particularly valid in this population of NSHW who seek solutions for work-related sleep disorders, stress and social difficulties.

### Study strengths and limitations

This study has limitations. First, the data collected came from only 10 research sites (AP-HP hospitals) in the Parisian area, and cannot be considered to be representative of all NSHW in France. Second, this study was focused only on a small proportion of NSHW. Physicians and pharmacists were not included as few work at night, and only in certain departments (e.g., accident and emergency). Third, participant recruitment was volunteer-based. We can therefore hypothesize that the persons interviewed were more inclined to talk about their consumption and addictions. To limit this bias, the investigators looked to recruit for a wide variety of profiles (age, sex, profession, department, and structure). Fourth, talking about alcohol and illegal drug consumption was often uncomfortable for the study participants, since this is still a taboo subject, especially in the healthcare setting. Fifth, NSHW believed there was a clear separation between their life during work and outside work. They did not consider that consuming PS outside of working hours could be influenced by their work. Consequently, it was difficult for some of the study participants to talk about their consumption of PS outside work.

The main strength of this exploratory qualitative study is that it is one of the first of its kind to focus on addictive behaviors and their determinants in NSHW, an understudied, hard-to-reach population due to their isolation. Our results can be used to orient future research on interventions for NSHW and the prevention of PS use for work-related reasons in this population.

## Conclusions and perspectives

The results of this study helped us create the self-administered questionnaire used for the ongoing ALADDIN quantitative study [59], which was proposed to all NSHW in all AP-HP hospitals. Its results will help us develop new research hypotheses about addiction management in this population.

To conclude, working at night and the stressful and stigmatized work environment affected NSHW mental and physical health. The use of PS in our study sample appeared to be for self-medication purposes, encouraged by social and professional environments, source(s) of stress, discrimination, and isolation.

Anti-stigma interventions in healthcare settings and screening for mental/somatic disorders in NSHW can help to reduce harmful self-medication behaviors, and improve hospital care, especially in the COVID-19 era.

## Supplementary Information

**Additional file 1.**

**Additional file 2.**

## Data Availability

The datasets generated and analyzed during the current study are not publicly available due to the French legislation on research involving the human person and the presence of potentially identifying or sensitive patient information but are available from the corresponding author on reasonable request.
