# Supplementary material for: Perceived stigma, substance use and self-medication in night-shift healthcare workers: a qualitative study
Source: BMC Health Serv Res. 2022 May 24;22:698. doi: 10.1186/s12913-022-08018-x (PMC9128768; doi:10.1186/s12913-022-08018-x)
Supplement: Supplementary file 1 — Additional file 1. [file 12913_2022_8018_MOESM1_ESM.pdf]

DINA – Night shift health workers substance use, a qualitative study

Framework - Qualitative study interview guide

Introduction

Within the framework of the DINA study, we are interested in the health of night shift professionals at the AP-HP, their quality of life, their use of substances, and their preferences regarding the management of certain addictive behaviors, particularly smoking.

This anonymous interview will be recorded, transcribed, and analyzed. Your participation and your answers will allow us to identify the main themes related to quality of life at work and the use of psychoactive substances among hospital night shift staff. It will also help guide the second part of this study, which consists of creating and distributing a questionnaire to all night shift workers about health, quality of life and addictions.

Socio-demographic data:

- Age
- Gender
- Profession and department
- Seniority in night shift work and current working rhythm (10h or 12h or 8h shifts)
- Family situation (children)
- Tobacco use (quantity and duration)

Questions :

- Work and Health:

How would you describe/define your work?

How would you rate your health?

Do you have any health problems that you think are related to your work?

Do you feel tired? Under what circumstances?

Do you ever feel anxious? Tell me about an experience where your work has caused you anxiety.

- Substances use:

(if smoker) In what context, how did you start smoking?

(if relevant) What motivated you to quit? Tell me how you went about quitting (let them describe the techniques and treatments). What are the advantages and disadvantages of these methods of quitting?

(if applicable) Why did you start smoking again last time?

(if applicable) How much do your colleagues smoke? What do you think of it?

(if applicable) What do your co-workers think about your smoking habits?

Have you ever experimented with certain types of drugs (e.g., cannabis, cocaine, amphetamines, morphine, etc.) even if you did not continue to use them afterwards?
(if relevant) Under what circumstances do you use alcohol (or other substances mentioned)?
(if relevant) How does working at night affect your use of alcohol (or other substances mentioned)?
(if relevant) What medications do you take?
What do your co-workers think about your drinking (or other substance use)?
Do you think you are addicted? (follow-up question: psychoactive substances, type of food (sugar), screens, internet, social networks).

- <u>Substance use and work:</u>

Have you had experience with caregivers using unauthorized substances in the workplace? What would you do if your colleague had a problem with alcohol or another substance?

How might night shift work impact addictions ?
Do you think that work can have an impact on substance use? Is this true for you? Tell me an experience where smoking (or other substance use) helped you decompress/escape/relax/sleep/keep you awake?
How did you manage your addictions? (if relevant)
How can we improve the management of addictions at APHP?
