# Supplementary material for: Perceived stigma, substance use and self-medication in night-shift healthcare workers: a qualitative study
Source: BMC Health Serv Res. 2022 May 24;22:698. doi: 10.1186/s12913-022-08018-x (PMC9128768; doi:10.1186/s12913-022-08018-x)
Supplement: Supplementary file 2 — Additional file 2. [file 12913_2022_8018_MOESM2_ESM.pdf]

Pseudonym	Tobacco	Alcohol	Cannabis	Sleeping pills
Thierry	Yes	Yes	Yes	No
Paul	No	Yes	Yes	No
Mohammed	Ex	Yes	Yes	Yes
Sarah	Yes	Yes	Ex	Yes
Franck	Yes	Yes	No	Yes
Aïcha	No	Yes	No	Yes
Téana	No	No	No	No
Guillaume	No	Yes	No	No
David	Yes	Yes	No	No
Max	Yes	Yes	No	Yes
Mireille	No	Yes	No	Yes
Sandrine	Ex	Yes	No	No
Lucile	Ex	Yes	No	No
Pierre	No	Yes	No	No
Dan	No	Yes	No	No
Mathilde	No	Yes	No	No
Benoît	No	Yes	No	No
Pierre Yve	Yes	Yes	No	No

Annex 2: Participants substance consumption
